# The National Congress of Surgery with international participation – the XXVII Edition, Sinaia, May 21-24th 2014

**Published:** 2014-09-25

**Authors:** S Constantinoiu

The National Congress of Surgery, one of the annual events to which the surgeons in all the surgery units in Romania gather to share the experience and to learn from one another (the other event being the National Conference) took place this year, in Sinaia, the most beautiful mountain resort, during 21-24th of May 2014. 

 “Casino Sinaia” International Conference Center offered through the great hall (the Theater) and four other halls, a sumptuous background, elegant and, in the same time, a proper place for the event. 

 What should be mentioned is the fact that this Romanian traditional medical scientific event has taken place under the high patronage of His Excellency, Victor Ponta, Prime Minister of Romania. 

 The main Congress organizer was the Romanian Society of Surgery (represented by prof. Eugen Bratucu, MD, President-in-Office, prof. Mircea Beuran, MD, Elected President and prof. Irinel Popescu, MD, Chief of the Clinic of Surgery and Liver Transplant of “Fundeni” Hospital and President of the Academy of Medical Sciences, and also members in the Congress Organization Committee) in collaboration with the Academy of Medical Sciences, Romanian Association of Hepato-Biliopancreatic Surgery and Liver Transplantation, Romanian Association of Endoscopic Surgery and other Intervention Techniques, Romanian Society of Emergency Surgery and Traumathology, Romanian Society of Breast Surgery, Romanian Society of Thoracic Surgery, Romanian Society of Pediatric Surgery, Romanian Society of Medical Assistants in Romania, Romanian Students’ Society of Surgery. 

**Fig. 1:**
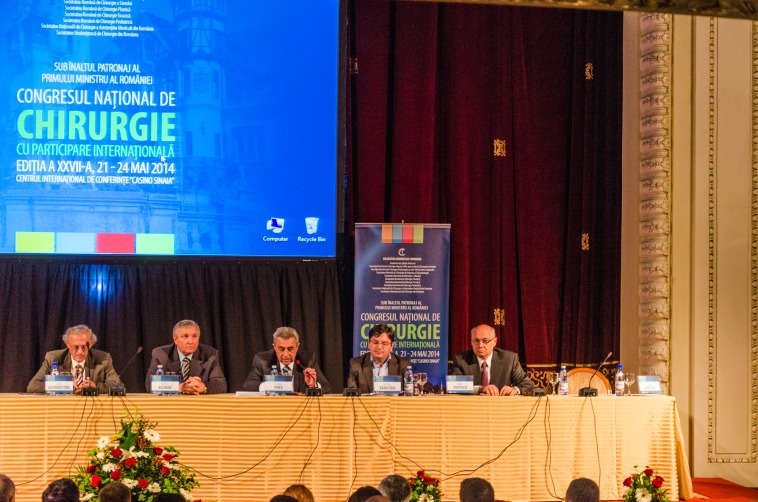


 What should be highlighted from the beginning is the high number of participants, surgeons from Romania and from abroad (over 1500) and over 300 students from all the universities in Romania, who successfully represented the Romanian Students’ Society of Surgery. 

Wednesday, the 21st of May 2014, was allocated to the eight pre-congress post university courses and, due to their importance and the special participation, we should shortly enumerate their content and the speakers, who are remarkable personalities in their field: 

 C1 – The surgical rectum anatomy; critical points in rectum surgery (prof. Fl. Filipoiu, MD, Assoc. Prof. V. Strâmbu, MD, Assoc. Prof. Dan Straja, MD); 

 C2 – Controversies in rectum surgery (S. Barbu, J. Jenkins, N. Yassin, G. Aniței, V. Scripcariu, Y. Vashist, J. Izbicki, V. Kepenekian, M. Adham, C. Duță);

 C3 – The minimally invasive rectum cancer surgery (prof. I. Popescu, MD, C. Vasilescu, Oana Stanciulea, C. Copăescu, V. Tomulescu, L. David);

 C4 – Causes of failure in the rectum cancer surgery (V. Scripcariu, I. Georgescu, I. Chiricuță, Al. Grigorescu);

 C5 – The surgery of the breast cancer (prof. Al. Blidaru, MD, prof. T. Burcoș, MD, C.I. Bordea, P.A. Achimaș Cadariu, S.C. Voinea, Dana Jianu);

 C6 – The multimodal treatment of breast cancer (D.C. Jinga, Dana Lucia Stănculeanu, A. Grigorescu, M. Savu, L. Stoleru, Mădălina Drăgănescu, Al. Blidaru, C.I. Bordea);

 C7 – The reconstruction of the breast after mammectomy (I.P. Florescu, I. Lascăr, T. Bratu, Al. Blidaru, N. Antohi, R. Ionescu, Z. Crăiniceanu) –Allergan Symposium;

 C8 – RSEST - MUSEC European Course; Ultrasound in trauma and emergency (M. Zago, A. Cassamassima, M. Marconi, M. Păduraru);

 What should also be mentioned is MUSEC European competence course, organized by the Romanian Society of Emergency Surgery and Traumatology - RSEST (prof. M. Beuran, M. Anastasiu, MD) “Ultrasound in trauma and emergency”, having prof. Mauro Zago, MD, from Bergamo responsible for the course and also the lecturers Andrea Cassamassima (Milano), Mateo Zarcani (Milano) and Mihai Păduraru (Tomelosso), all from Italy, a course to which subscriptions have been made a lot of time before, due to the limited number of participants given by the type of the workshop. 

 This year’s congress has guided itself on two main themes: “The complex treatment of rectum cancer” and “Breast cancer. Complex treatment. The reconstructive surgery of the breast”. 

 The scientific program was not limited only to these two themes and (due to the inherent monotony which appears in the monothematic congresses), has contained and varied also video laparoscopic surgery, hepato-biliopancreatic surgery, endocrine eso-gastric surgery. 

 Fully, over 500 papers were presented in the 5 halls of the congress which benefited from the necessary modern infrastructure. 

 The festive opening of the Congress took place on Thursday evening, the 24th of May, in the presence of the Ministry of Health, His Excellency Nicolae Bănicioiu (specialized in dentistry), the mayor of Bucharest, prof. Sorin Oprescu, MD (specialized in surgery), prof. Florian Popa, MD (specialized in surgery and also the vice-president of the Health Commission of the Senate of Romania), prof. Vasile Astărăstoaie, MD (president of the College of Physicians in Romania), prof. Mircea Beuran, MD (elected president of RSS), prof. Eugen Brătucu, MD (RSS executive president was not able to take part in the event due to medical reasons). In their presentations, the members of the presidium have highlighted the difficulty of undertaking a medical and surgically correct and decent act for the patient, taking into account the crisis in the Romanian medical system, suggesting diverse solutions, among which, the one of Senator, prof. Florian Popa, MD, regarding the liberalization of the physician profession in Romania. 

 The way of development of the congress was based on novelty, according to a European model, less mini oral papers being selected, the other ones migrating to posters, the “state of the art” presentations and round tables having priority, with interdynamic development of the discussion themes, having the expected success. 

 The rectum surgery was one of the main themes of the congress and raised a special interest. The following sessions led by prof. Ştefan Georgescu, MD, prof. Traean Burcoş, MD, assoc. prof. Cătălin Vasilescu, prof. Lazăr Fulger, MD, prof. Adrian Magyar, MD, assoc. prof. Victor Strâmbu, MD, Cătălin Copăescu, MD, prof. Silviu Constantinoiu, MD (the last one with an accent on colorectal cancer) should be mentioned. The round tables on the same subject were mediated by prof. C. Copotoiu, MD, prof. Tr. Pătraşcu, MD, prof. V. Scripcariu, MD, assoc. prof. Dan Straja, MD, prof. Mircea Beuran, MD, as well as the one regarding radiochemotherapy in rectum cancer (prof. Cristian Chiricuţă, MD), or colonic surgery (prof. Radu Palade, assoc. prof. Adrian Miron, assoc. prof. Laurenţiu Beluşică) and have been the most interesting for the participants. 

 The rectum minimally invasive surgery (thoroughly approached in the pre-congress course coordinated by prof. Irinel Popescu, MD) has raised a special interest, like the other themes did, such as the total resection of the mesorectum, the anatomo-pathological examination of the resection part, the complications of rectum surgery. 

The sessions allocated to the breast surgery have completed the themes approached in the pre-congress courses (the breast cancer surgery, the multimodal treatment of breast cancer, the reconstruction of the breast post cholecystectomy) highlighting the ones led by prof. Eugen Târcoveanu MD, prof. Ştefan Neagu, MD, prof. Ion Aşchie, MD, assoc. prof. Iulian Brezean, prof. Alex. Irimie, MD, prof. M.R. Diaconescu, MD, prof. Dan Ungureanu, MD, prof. Cristian Lupaşcu, MD. Totally, there were 33 papers and “state of the art” conferences which approached the theme of breast cancer conservative surgery (prof. Al. Blidaru, MD), sentinel limph node in breast cancer (C. Bordea, MD), multidisciplinary treatment of breast cancer (prof. Alexandru Irimie, MD), breast oncoplastic surgery, breast cancer surgery with liver metastases, prognosis of breast cancer, Volkman acute carcinomatous mastitis. The round tables have perfectly completed the theme of breast gland pathology: 

 - The oncologic surgery of the breast (coordinator prof. Al. Blidaru, MD);

- The actual, diagnostic and therapeutic standards in breast gland cancer (Fl. Rădulescu); 

- The present coordinates in breast reconstruction after oncologic resections (prof. I. Lascăr, MD, prof. Al. Blidaru, MD prof. T. Bratu, MD).

 Two sessions were allocated to hepato-biliopancreatic surgery, summing 26 papers, remarking the ones dedicated to liver tumors (prof. Vladimir Hotineanu, MD), pancreatic tumors (prof. A. Olah, MD), bilio bronchial fistulae (T. Ivanov), Klatskin tumors, liver resections (prof. N. Vladov, MD, prof. M. Miliacevic, MD), cephalic duodenopancreatectomy (prof. C. Copotoiu, prof. C. Iancu, MD), pancreatic neuroendocrine tumors (prof. Cr. Lupaşcu, MD), liver transplant (prof. I. Popescu, MD). 

 Pediatric surgery (coordinator prof. C. Sabetay, MD) discussed the disorders due to the migration of the testicle in children, hydatid cysts and the treatment of congenital diaphragmatic hernias in children. 

 The minimally invasive surgery, present mostly in ARCE symposium, has remarked, as usually, through the robotic surgery presentations (assoc. prof. Cătălin Vasilescu), „bridge to surgery” in colorectal cancer (assoc. prof. Cătălin Copăescu, MD), minimally invasive surgery of the big hiatal hernias (prof. V.V. Grubnik, MD). 

 The thoracic surgery session, coordinated by prof. Ioan Cordoş, MD, discussed the minimally invasive thoracoscopic approach, emergency thoracotomy, complex resections of the aerodigestive tract, thymectomy and laparoscopic splanhnicectomy. 

 On Saturday, the 24th of May (the last day of the congress, far from lacking a wide participation) continued with a session dedicated to gastric surgery (moderators prof. Octavian Unc, MD, prof. Nicolae Iordache, MD, assoc. prof. Valentin Grigorescu, MD, S. Păun, MD), the papers from the “Sf. Maria” General and Esophageal Surgery Clinic being remarked (the mutational analysis in GIST, the cancer of the cardia), total gastrectomy versus subtotal gastrectomy (prof. C. Copotoiu, MD), GIST (acad. N. Ghidiriu). The esophagus surgery session (moderators: prof. S. Constantinoiu, MD, prof. I. Cordoş, MD, prof. Dan Sabău, MD) discussed about high interest and technical problems: minimally invasive esophagectomy (prof. N. Dănilă, MD), therapeutic strategy in thoracic esophagus squamocellular (prof. S. Constantinoiu, MD), the treatment of malignant eso-bronchial fistula through a metallic stent expendably covered (prof. Gabriel Dimofte, MD). 

 The endocrine surgery session (moderators: prof. M.R. Diaconescu, MD, assoc. prof. V. Strâmbu, MD, prof. C. Diaconu, MD) dealt with modern problems regarding parathyroidectomy, thyroidectomy and intraoperative monitorization of the recurrent nerve (R. Popescu, MD, and the group from Rm. Vâlcea). 

 Last (but not least), the session “Surgery of the abdominal wall” (moderators: prof. D. Mogos, MD, prof. Costel Şavlovshi, MD, prof. N. Dănilă, MD, M. Pop, MD, F. Gavrilaş, MD) was very instructive. 

 This review of the congress papers would not be complete if we did not mention the session dedicated to the students’ papers, who were present in a high amount (moderators: M. Marincaş, MD, Alexandra Bolocan, MD and Dan Păduraru, MD), and who presented papers elaborated in the students’ scientific circles in Romania and at the professors’ guidance. The video film session closed the last day of the congress papers. 

 What should be noticed is the high amount of students and medical assistants taking part in the event, as well as the value of the 264 posters. In the generous setting of the Casino in Sinaia, the medical devices and drugs companies had a functional exposing location. 

**Fig. 2:**
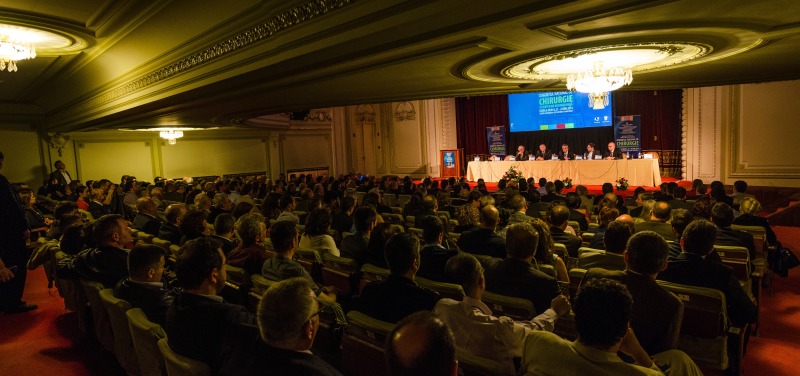


 On the occasion of the congress, an editorial event also took place in one of the breaks between the sessions, in the presence of a numerous public, physicians and students: 

 - The launching of the monography “Acute pancreatitis – clinical-paraclinical approach based on determinant factors” (authors: D. Cochior, S. Constantinoiu, “Carol Davila” University Press, Bucharest, 2014), a paper that makes a connection between Atlanta 1992 criteria and Bologna 2012 criteria. The short speeches of the authors of the two prefaces, prof. Florian Popa, MD, prof. Eugen Târcoveanu, MD, as well as the ones of two authors have highlighted the strong points of the paper, which is desired to be useful to all the physicians. 

 - In the same time, “Journal of Surgical Science” for the students and young physicians has been launched; it will appear in English at the initiative of Alexandra Bolocan, MD and şi Dan Nicolae Păduraru, MD, Honorary Editor – acad. Ioanel Sinescu, rector of “Carol Davila” University Press, Editor-in-Chief – prof. S. Constantinoiu, MD, Managing Editor prof. Florian Popa, MD, having an Editorial board made up of students and young physicians in Romania, as well as abroad. The magazine proposes to be the national sign of the students’ scientific circles from all the university centers. Moreover, it should be mentioned that these papers appeared in the generous space of “Carol Davila” University Press in Bucharest, General Manager, assoc. prof. dr. Victor Lorin Purcărea. 

 Moreover, the “Surgery Course for the 4-5th years of study” was elaborated by the professors in the 10th Surgery-Laboratory Department, where the Head of Department is prof. Mircea Beuran, MD. 

 In the RSS General Gathering, important modifications have taken place in the structure of the editorial board of the great magazine of the Romanian surgery, “Chirurgia”, the editor-in-chief becoming RSS executive president, validated in the congress, prof. Mircea Beuran, MD, and the editorial secretary being prof. Silviu Constantinoiu, MD, also elected president of RSS. Moreover, 7 vice-presidents of RSS have also been elected, the general secretary (being the same, assoc. prof. Dan Straja, MD, as well as the treasurer of the Society, assoc. prof. Victor Strâmbu, MD), the auditing committee and RSS Directory Committee. 

 We consider that from all points of view, the National Surgery Congress this year has been a real success, reiterating once more the force of the professional organization of the Romanian surgeons, the foreign guests arguing the value of the debates and the presentations, being impressed by the value of the Romanian surgery. 

 Prof. Silviu Constantinoiu, MD

 Director of the Excellency Center of Esophagus Surgery 

 Head of the General and Esophageal Surgery Clinic 

 “Sf. Maria” Clinical Hospital 

 “Carol Davila” University of Medicine and Pharmacy, Bucharest 

